# Risk factors for physician burnout: a perspective from Tanzania

**DOI:** 10.11604/pamj.2022.41.298.31055

**Published:** 2022-04-13

**Authors:** Shweta Iyer, Shahzmah Suleman, Yuqing Qiu, Shari Platt

**Affiliations:** 1 New York-Presbyterian, Weill Cornell Medicine, New York, United States of America,; 2 Bugando Medical Centre, Mwanza, Tanzania

**Keywords:** Burnout, wellness, Tanzania, global health

## Abstract

**Introduction:**

while physician burnout has been studied in high-income countries, more research is necessary on burnout in lesser-income regions such as Tanzania. This study aimed to determine levels of burnout in Tanzanian physicians and to understand the contributing risk factors for burnout in this region.

**Methods:**

the Maslach Burnout Inventory (MBI-HSS) was adapted to assess burnout in Tanzanian physicians. Utilizing a cross-sectional design, we studied two distinct cohorts: 1) Emergency Medicine (EM) trained physicians in Tanzania and; 2) specialists at Bugando Medical Centre. We surveyed demographic, personal, and workplace data to identify risk factors for burnout.

**Results:**

seventy-seven percent (30/39) of Tanzanian EM providers and 39% (37/94) of Bugando specialists completed the survey. We identified burnout in 67% of Tanzanian EM providers and in 70% of specialists at Bugando. Burnout risk factors in EM physicians included dissatisfaction with career choice, considering switching institutions, working in an urban setting, inadequate coverage for emergencies/leave, and financial housing responsibilities. In Bugando specialists, risk factors were unnecessary administrative paperwork, working overnight shifts, pressure to achieve patient satisfaction or decrease length of stay, meaningful mentorship, and not having a close friend/family member die.

**Conclusion:**

this study reports a high prevalence of burnout in Tanzanian physicians. Risk factors for burnout were multifactorial but mainly related to institutional and workplace constituents. Targeting these risk factors provides opportunities to boost physician wellness and guides important areas for future research in this African region.

## Introduction

While burnout has been extensively studied in high-income regions, much less is known about physician burnout in lesser-income settings such as African countries. Per the limited literature, burnout seems to be on the rise in Africa and some of the correlates are similar to those identified in the United States of America such as heavy workload, poor work-life balance, lack of institutional support, and poor teamwork [1,2]. However, the existing studies do not explain contributing factors in detail, therefore leaving unclear how to best mitigate potential risk factors for burnout. Our research aimed to determine the prevalence of burnout in Tanzanian physicians and to investigate contributing risk factors for burnout in this region.

## Methods

We conducted a survey study utilizing a cross-sectional design with mixed methods analysis. The survey consisted of two components: 1) The Maslach Burnout Inventory Human Services Survey (MBI-HSS); a validated tool to measure burnout, and; 2) a demographic survey to include all possible risk factors associated with burnout.

**Maslach burnout inventory human services survey:** the MBI-HSS is the most commonly used instrument for measuring burnout in physicians [1]. We used a modified MBI-HSS with questions adjusted to account for cultural differences between Africa and the United States of America based on expert opinion and guidance by the Tanzanian co-investigator, and as had been done in a prior study [1]. Both survey versions are included in Annex 1.

**Demographic survey:** the second portion of the survey was developed in a rigorous manner after extensive item generation and reduction, along with pilot testing to ensure that all burnout risk factors were included and assessed. Initially, supplemental factors were chosen via review of prior burnout surveys studied and employed in publications in the United States of America. A thorough literature review was then performed to assess burnout risk factors unique to Eastern Africa. These were then presented to and discussed with key stakeholders in Tanzania to generate relevant supplemental questions. Items were removed or included as relevant, and pilot tested multiple times on both Cornell and Bugando content experts and colleagues to ensure ease of comprehension and generalizability. Once created, the adapted surveys were again presented and discussed with Tanzanian physicians to ensure that unique burnout risk stressors were captured in culturally appropriate wording and relevant to the local study population. Trialing the adapted surveys for clarity of language and time on task allowed for further analysis and modification. Both questionnaires were piloted prior to formal distribution. Throughout this process, the surveys were refined and revised based on these assessments and feedback. The final surveys included the modified MBI-HSS and the section on demographic factors.

**Survey distribution:** over the past decade, nearly forty physicians currently working in major regions of Tanzania have received specialty training in emergency medicine (EM). Other specialties have been training residents for much longer, and at Bugando Medical Centre in Mwanza, Tanzania, there are almost 100 specialists in various fields. Subjects eligible for inclusion consisted of two separate cohorts: 1) physicians trained in EM throughout Tanzania, and; 2) physicians in all specialties working at Bugando Medical Centre. The surveys were created using Qualtrics software and were distributed to EM physicians via a text on the WhatsApp application and Bugando specialists via email, using a reusable link unable to track identifying information of respondents. The survey was sent to all eligible subjects in both study cohorts weekly for two months during August and September of 2020 (seven times total) to ensure the highest possible response rate. We excluded subjects if they started but failed to complete at least 90% of the survey, and/or had all 3 MBI-HSS scores=0.

**Outcome definitions:** the MBI-HSS measures burnout on three separate subscales: emotional exhaustion (EE), depersonalization (DP), and personal accomplishment (PA). We included varied definitions of burnout based on prior literature. Our primary outcome was burnout defined by scoring in the high-degree range on any one of the three subscales of the MBI-HSS (≥27 EE, ≥10 DP, or ≤33 PA). Alternate definitions of burnout included scoring in the high-degree range on all three subscales, scoring in the high-degree range on either the EE or DP scales, and answering positively to a 2-item survey used as a surrogate for EE and DP: “I feel burned out from my work” and “I have become more callous toward people since I took this job”.

**IRB approval:** IRB approval was sought and obtained from both institutions, Weill Cornell Medicine and Bugando Medical Centre, and the Tanzanian National Institute for Medical Research (NIMR).

**Analysis:** the outcome for the study was the presence of burnout. Demographic information and potential risk factors from the questionnaires were summarized for all providers. For categorical variables, counts and percentages of patients in each group along with p-value from chi-squared or Fisher´s exact test were reported as appropriate. For numerical variables, mean (SD) or median (IQR) along with p-value from two-sample t-test or Wilcoxon rank-sum test were reported as appropriate. To detect the risk factors, univariate logistic regression with presence of burnout as an outcome variable was performed for items with p-value <0.15 in the bivariate descriptive analysis mentioned above. Odds ratios along with 95% confidence intervals and p-values from univariate logistic regression were reported. Given the sample size and the exploratory purpose of the study, p-values were not adjusted for multiplicity. Based on the univariate regression result, variables were considered as associated with getting burnout at significance levels of 0.05 and 0.1. Analyses were implemented for the emergency medicine provider´s cohort and the specialist´s cohort separately. Demographic survey questions were measured on Likert scales and categorized into dichotomous outcomes. Questions with optional free text responses were redefined as aggregated variables (i.e. simplified to “yes” or “no” for the purposes of analysis). All analyses were performed in R version 4.0.2 (2020-06-22).

## Results

**Figure 1:**
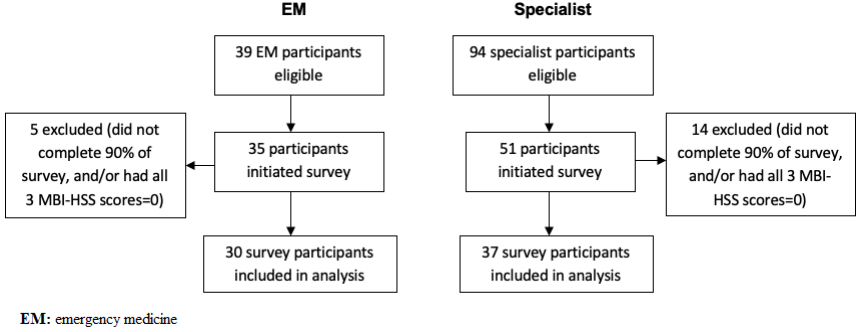
patient enrollment flow diagram

Surveys were completed by 77% (30/39) of Tanzanian EM providers and 39% (37/94) of Bugando specialists (Figure 1). Demographic data for both study cohorts is shown in Table 1. Per the primary burnout definition of scoring positively on any one subscale of the MBI-HSS, we found that 67% (20/30) of Tanzanian EM providers and 70% (26/37) of Bugando specialists had burnout. Table 2 displays the burnout rates for both study cohorts based on all definitions for burnout.

**Table 1:** demographics

		Burnout (EM)	No burnout (EM)	Burnout (specialist)	No burnout (specialist)
Median age (IQR)		36 (34.75, 37.25)	37 (36, 39.5)	37 (35, 39)	36 (35, 44)
Gender	Female	6 (67%)	3 (33%)	8 (67%)	4 (33%)
	Male	13 (65%)	7 (35%)	17 (77%)	5 (23%)
Marital status	Married or domestic partner	15 (65%)	8 (35%)	22 (73%)	8 (27%)
	Single, never married	3 (60%)	2 (40%)	3 (100%)	0 (0%)
	Divorced/other	1 (100%)	0 (0%)	0 (0%)	1 (100%)
Parental status	Children live in home	10 (59%)	7 (41%)	18 (72%)	7 (28%)
	Children live out of home	4 (100%)	0 (0%)	1 (100%)	0 (0%)
	No children	5 (71%)	2 (29%)	5 (71%)	2 (29%)
Prior career	No	17 (63%)	10 (37%)	22 (73%)	8 (27%)
	Yes	3 (100%)	0 (0%)	1 (100%)	0 (0%)
Years of practice	<5 years	13 (65%)	7 (35%)	13 (72%)	5 (28%)
	5 - 10 years	6 (75%)	2 (25%)	6 (75%)	2 (25%)
	>10 years	0 (0%)	1 (100%)	3 (75%)	1 (25%)
Work environment	Rural/suburban	2 (33%)	4 (67%)	N/A	N/A
	Urban	17 (74%)	6 (26%)	N/A	N/A
Median clinical hours per week (IQR)		49 (34.5, 62.5)	48 (41.25, 60)	50 (46, 60)	55 (45, 71)
Non-clinical work	Yes	17 (63%)	10 (37%)	20 (74%)	7 (26%)
	No	1 (100%)	0 (0%)	2 (67%)	1 (33%)
Wellness program	Yes	6 (60%)	4 (40%)	8 (80%)	2 (20%)
	No	13 (68%)	6 (32%)	12 (67%)	6 (33%)

**Table 2:** burnout rates based on study definitions

	Burnout (EM)	No burnout (EM)	Burnout (specialist)	No burnout (specialist)
Any one of the three subscales	20 (67%)	10 (33%)	26 (70%)	11 (30%)
ALL three subscales	3 (10%)	27 (90%)	5 (14%)	32 (86%)
Either the EE or DP	18 (60%)	12 (40%)	22 (59%)	15 (41%)
Two-item survey: “I feel burned out from my work” and “I have become more callous toward people since I took this job”	11 (37%)	19 (63%)	16 (43%)	21 (57%)

EM: emergency medicine; EE: emotional exhaustion; DP: depersonalization

In a logistic regression analysis, for Tanzanian EM providers, factors of: not feeling satisfied with a career choice in emergency medicine; feeling that the respondent would opt out of working for the current institution and move to another institution; primarily working in urban communities; not having adequate coverage for unplanned emergencies or personal leave; and having financial responsibilities regarding housing were found to be associated with burnout (Table 3). For specialists at Bugando, factors associated with burnout included: having unnecessary administrative paperwork; working overnight shifts; having institutional pressure to achieve patient satisfaction; pressure to decrease length of stay for patients; having meaningful mentorship; and not having a close friend or family member die (Table 3).

**Table 3:** logistic regression analysis for burnout risk factors

Cohort	Risk factors	OR (95% CI)	p-value
EM	Primarily working in urban communities (urban vs rural/suburban)	5.67 (0.88, 49.39)	0.079+
	No adequate coverage for unplanned emergencies or personal leave	5.67 (0.88, 49.39)	0.079+
	Not satisfied with your career choice in emergency medicine	10 (1.45, 203.74)	0.045*
	Felt in the last year that you may opt out of working for the current institution and move to another	8.5 (1.39, 74.09)	0.029*
	Currently have financial responsibility in housing	4.5 (0.93, 24.97)	0.069+
Specialist	Work overnight shifts	7.5 (0.97, 158.55)	0.09+
	There is unnecessary administrative paperwork	6.67 (1.17, 46.47)	0.039*
	Pressured by your institution to achieve patient satisfaction	11.4 (1.18, 260.86)	0.053+
	Pressured to decrease length of stay for your patients	5 (0.91, 32.78)	0.072+
	Have meaningful mentorship	5.57 (0.99, 45.63)	0.068+
	Had a close friend or family member die	0.18 (0.03, 1.05)	0.059+

+p<0.1; *p<0.05; EM: emergency medicine

## Discussion

This cross-sectional study displayed a moderately high prevalence of burnout in Tanzanian physicians. A recent study from Central Africa similarly showed that almost 50% of surveyed practitioners manifested burnout, displaying the magnitude and scope of this problem on the African continent [3].

In the EM physician cohort many of the risk factors we found to be associated with burnout, such as primarily working in urban communities, not having adequate coverage for unplanned emergencies or personal leave and having financial responsibilities for housing, all relate to workplace and job-related factors. Similarly, Bugando specialist risk factors for burnout such as having unnecessary administrative paperwork, working overnight shifts, having institutional pressure to achieve patient satisfaction or decrease length of stay for patients, and having meaningful mentorship, encompass institutional factors as well. These findings highlight clinical, quality, and educational work-related responsibilities as important contributors to burnout. Prior research in sub-Saharan Africa similarly showed that the workplace environment was the primary factor associated with burnout, including analogous reasons such as career dissatisfaction, working conditions, support staff, and perceived workload [4]. A recent prior study in South Africa demonstrated that time-related workplace pressures, a lack of professional growth options, and a lack of supportive organizational supervision were positively associated with burnout [5]. While similar to data reported from the USA [6,7], this contrasts from our findings since “having meaningful mentorship” was found to be positively associated with burnout. We hypothesize a distinction between supportive supervision and meaningful mentorship, as meaningful mentorship may confer high academic expectations on the physician.

Examining the specialist risk factors raises some notable comparisons between Tanzania and the United States. Similar to our finding of unnecessary administrative paperwork as a risk factor for burnout, the electronic health record has been found to contribute to burnout in North American physicians, displaying a parallel between the settings despite differing resources [8]. Working long hours and overnight calls has also been shown to contribute to physician burnout in the United States of America, as in Tanzanian specialists [9]. Targeted workplace interventions in the United States of America have shown marked improvements in physician wellness [10]. Consequently, these institutional components likely have implications for future wellness initiatives in Tanzania as well.

The sociocultural differences between Tanzania and other countries are worth highlighting. For example, having a close friend or family member die, a personal factor, was found to be protective of burnout. While counterintuitive, it may reflect that some Tanzanian providers care for members of their extended family and subsequently bear the costs of treatment along with other logistics. When the family member passes away it may relieve these burdens and reduce burnout. Sociocultural and personal factors for burnout in Tanzanian physicians are important and need further investigation.

**Limitations:** this study was limited by small sample size in the EM cohort, despite high participation. Expanding this study to other countries in Eastern Africa may identify additional significant risk factors and improve generalizability. Another limitation was a low response rate in Bugando specialists. Although we distributed the survey multiple times, future research initiatives may include incentives to enhance study participation. Nevertheless, our results align with prior literature targeting the institutional constituent in burnout and provide groundwork for future study.

## Conclusion

This study reports a high prevalence of burnout in Tanzanian physicians. Risk factors for burnout were multifactorial but mainly related to institutional and workplace constituents. These findings also highlight societal and cultural differences between physicians in Tanzania compared to those in the United States of America. Targeting these risk factors provides opportunities to boost physician wellness and guides important areas for future research in this African region.

### What is known about this topic

- *Physician burnout is prevalent in high-income countries;*
- *Physician burnout is influenced by both personal and institutional factors*.

### What this study adds

- *Physician burnout is high in Tanzania;*
- *Institutional risk factors contribute to physician burnout in Tanzania*.

## Annex

**Annex 1**: survey: burnout in Tanzania for EM (PDF-160 Kb)
